# Living the Full Catastrophe: A Mindfulness-Based Program to Support Recovery from Stroke

**DOI:** 10.3390/healthcare8040498

**Published:** 2020-11-19

**Authors:** Lori A. Gray

**Affiliations:** Holistic Health Programs, Western Michigan University, Kalamazoo, MI 49008, USA; lori.gray@wmich.edu

**Keywords:** mindfulness, mindfulness-based stress reduction, mindfulness-based stroke recovery, stroke recovery, social support for stroke survivors, medical education, stroke rehabilitation

## Abstract

Decades of research suggest that Mindfulness-Based Stress Reduction (MBSR) training supports a greater capacity to live with chronic medical conditions and contributes to lowering stress levels. This paper introduces a model for a Mindfulness-Based Recovery from Stroke (MBRfS) for promoting stroke recovery, informed by the lived experience of the author (a stroke survivor and certified MBSR instructor), the research literature regarding MBSR training, and the specific challenges of stroke recovery. Four themes emerged from the autoethnographic analysis that informed the proposed model: Readiness to accept the stroke event and the acquired brain injury, navigating uncertainties of stroke recovery with awareness and self-responsibility for outcomes, trusting the inherent wisdom of the body as a stroke recovery “teacher”, and increased capacity to integrate complex emotions with self-compassion, and a sense of wholeness. A four-component MBRfS model is offered, which consists of an integration amongst a modified MBSR framework, emergent attitudinal themes, and insights from the autoethnographic vignettes. The MBRfS model offers a path for providing participants with a supportive experience within stroke recovery. Recommendations and suggestions for future studies are offered to support the development of MBRfS for stroke survivors and their caregivers, as well as contributing to healthcare providers.

## 1. Introduction

### 1.1. Mindfulness-Based Stress Reduction

Mindfulness-Based Stress Reduction (MBSR) is an eight-week, evidence-based protocol created by Dr. Jon Kabat-Zinn in 1979 as part of the Stress Reduction Clinic at the University of Massachusetts Medical School. Mindfulness skill training is based on Kabat-Zinn’s landmark book Full Catastrophe Living, where he operationally defines mindfulness as “the awareness that arises by paying attention on purpose, in the present moment, and non-judgmentally” [1] (p. iiiv). The story that informs the title of the landmark book comes from the novel Zorba the Greek. Kabat-Zinn describes a scene where Zorba the Greek and his companion are talking and his friend asks “Zorba, have you ever been married?”, to which Zorba replies, “Am I not a man? Of course, I’ve been married. Wife, house, kids…the full catastrophe!” [1] (p. liii). The story that served as the inspiration for the book’s title is not a reference to Zorba complaining, rather it is a testament to the attitudes of mindfulness, of embodying the acceptance of life (with its inevitable twists and turns), and choosing to experience joy and not be defeated by sorrow or hardships. This is part of the foundational tenets of MBSR. 

Initially, Kabat-Zinn created the MBSR program for patients undergoing treatment for whom mainstream medicine was not effective or sufficient. People with conditions such as chronic pain, cardiac disease, and symptoms exacerbated by stress were referred to the program. Physician confidence in MBSR increased as patients expressed positive changes in symptomatology [2]. 

The medical field has further demonstrated openness to the value MBSR provides as supportive training for healthcare providers. A recent systematic review from Lamothe et al. [3] suggests that MBSR training is associated with lowered stress levels, increased psychological health, and greater empathy in healthcare professionals. In addition to empirical evidence on MBSR’s efficacy in reducing both perceived stress levels and the impact of stress in patients and providers, there is emerging research that mindfulness meditation training specifically reduces psoriasis [4], chronic pain [5], restless legs syndrome [6], premenstrual dysphoric disorder [7], hot flashes related to menopause [8], and symptoms related to cancer, heart disease, depression, and anxiety [9]. 

For all these benefits, MBSR training is not touted to be a panacea or “cure-all”. It has been utilized as an adjunct mind–body therapy for medical treatment and psychotherapy. MBSR is an educational intervention and does not adhere to a group psychotherapy model, though the experience is considered therapeutic. Therefore, MBSR is not considered a medically necessary treatment, and most health insurance companies in the United States do not cover the costs of participating in MBSR training. Often referred to as “participatory medicine”, MBSR offers genuine hope and opportunities for well-being to many who are considered lost causes by physicians or those who fall between the cracks of traditional medicine [10].

A variety of psychological issues can be addressed through MBSR training in conjunction with psychotherapy and other modalities, but MBSR is not a substitute for individual or group psychotherapeutic treatment. It may be valuable in overcoming anxiety [11], preventing depressive relapse by reducing cognitive rumination [12], nicotine addiction and impulse control regulation [13], and recovery from trauma [14]. Contemplative neuroscience research suggests significant potential for the application of MBSR in shaping the structure and function of the brain, offering compelling future hopes for putting the mind–body connection to work in epigenetics, regulating the immune system, lowering inflammatory activity, and influencing autoimmune activity [15].

A recent meta-analysis of published empirical evidence in non-clinical, controlled studies, firmly establishes MBSR training as an effective mind–body intervention [10]. Experimentation on mindfulness meditation is further exploring the effects of mindfulness meditation on “re-wiring” the brain through the potential of neuroplasticity. Zeidan et al. [16] demonstrated that mindfulness meditation, practiced for only three days, was associated with consistent changes in brain scans compared to those who were guided through three days of “sham” mindfulness meditation, where general relaxation techniques were taught instead. This study was one of the first of its kind to incorporate the impact of the placebo effect within its analysis of mindfulness. The changes in brain scans within the mindfulness meditation group indicated more communication and activity in the parts of the brain that mediate stress reactivity, as well as areas of the brain that support focus and states of calm [17]. As the human brain carries the potential to shape and shift itself to fit our needs to varying degrees throughout adulthood, mindfulness meditation may be an avenue for harnessing this capacity for greater health and healing. Mindfulness meditation is also related to an increase in cortical thickness and changes in the right anterior insula, as assessed by functional MRI scans [18]. These areas are associated with sensory processing, focused attention, and interception.

### 1.2. Prevalence of Stroke and the Challenges for Recovery from Stroke

Current statistics indicate that one in every six people will experience a stroke, worldwide. As a leading cause of death and disability, approximately 80 million people have experienced and survived a stroke [19]. A stroke is a medical event in which the brain is deprived of oxygen, creating an acquired brain injury, the impact of which is dependent on the location and severity of the stroke event. There are many facets to the process of stroke recovery. Beyond the medical aspects, there are also numerous social, emotional, financial, vocational, and psychological impacts. Recovery from stroke is a lifelong endeavor to cope and make peace with a life-altering health crisis. It often requires a steep learning curve that can be very challenging to navigate for both the survivors and their loved ones [20].

There are common acute physical outcomes for the majority of stroke events. Typically, there is physical disability, often paralysis in the stroke-affected side, as well as loss of proprioception, loss of physical sensation related to touch, and a variety of stroke-related pain [21]. Stroke survivors also experience cognitive difficulties such as memory and attentional deficits, sensory integration difficulties (sometimes referred to as sensory flooding), and speech or language difficulties (aphasias) [22]. 

The stress related to the trauma and sudden physical losses and impairments often leads to heightened levels of depression, anxiety, and emotional distress [23]. The confluence of physical and psychological factors can create an ongoing chain of social and environmental stressors, such as isolation, financial burden, temporary or permanent loss of employment, and cultural stigma [24]. Stroke survivors may feel a deep loss of identity [25] while experiencing intense grief in the midst of the critical need to be motivated and fully engaged in their recovery. This may present a daunting life scenario with no clear end in sight. The stroke event for each survivor is a unique experience, and the path toward recovery is often an arduous one lacking a clear roadmap. One unifying and supportive perspective may be to see the inherent dignity and strength in each stroke survivor, even as they grieve the losses and navigate the enormous challenges of stroke recovery. This perspective points toward resiliency skills, or what is typically referred to as the ability to “bounce back” from setbacks [26]. 

There is a relatively small amount of research literature focusing on the impact of resiliency skills in successful stroke recovery. However, the consensus is clear, individuals who are assessed to be higher in psychological resiliency also report greater gains over time in recovery studies [26,27,28]. Therefore, stroke rehabilitation should also include interventions that focus on prioritizing, enhancing, and building resilience in stroke survivors. One pathway to building resilience, while also lowering stress, is to participate in a mindfulness-based skills training.

### 1.3. Mindfulness Skills to Support Stroke Recovery

A modest number of studies have sought to evaluate aspects of mindfulness and related mind–body interventions as a support in stroke recovery [29,30]. Yoga, and other mind–body movement practices, have been shown to be supportive in stroke recovery. Recent studies [31,32] provide exploratory evidence to support that yoga and mindful movements support improved aspects of motor and cognitive function for stroke survivors. Feasibility studies and systemic analyses suggest that with appropriate modifications and accessibility, yoga is perceived and experienced by stroke survivors as a valuable part of their recovery plan [33,34]. 

Additional studies have offered piloted initiatives with results that support the value of mindfulness training in reducing some of the more prominent physical challenges for stroke survivors, including the value of group training and social support. Two weeks of mindfulness meditation exercises were shown to improve post-stroke spasticity [35]; however, the study did not follow-up to assess lasting effects post-intervention. Initial aphasia studies using mindfulness training offer encouraging outcomes, but need further study and attention [36,37]. Mental and physical fatigue is another common difficulty post-stroke, usually due to acquired brain injury (ABI). Initial research for acquired brain injury patients suggests that mindfulness meditation may significantly relieve the impact of ABI-related fatigue [38,39]. 

A feasibility study to consider adaptations of mindfulness meditation delivery suggests that participants may prefer the flexibility to practice independently for short periods of time [40]. Another exploratory inpatient study with stroke survivors, consisting of a two-week daily mindfulness meditation, was found to significantly improve stroke survivors’ mindfulness and comfort levels [41]. Depression is very common in stroke survivors, and a pilot study made the case for the value of MBSR training to reduce depression in this vulnerable population [42]. The results of the study were inconclusive but encouraging for further investigation. 

### 1.4. Rationale for MBSR Adaptation and Introduction of MBRfS Model

Academic research demonstrates that mindfulness skills support effective mind–body interventions for stress reduction, as well as coping with various health conditions. Building upon Kabat-Zinn’s [1] insights, the MBSR, and the author’s direct experiences of stroke recovery, this preliminary study sought to accomplish the following objectives:

1. Review the literature to identify, frame, and affirm the beneficial effects of mindfulness skills training within the context of stroke recovery, as this opportunity has not been widely reviewed.
2. Explore the existing literature and personal documents for unique challenges and adaptations to consider when offering a mindfulness-based intervention for stroke survivors, their caregivers, and those offering healthcare to this population.
3. Propose a series of adaptations to Kabat-Zinn’s [1] MBSR framework to create a mindfulness-based curriculum tailored to stroke recovery.
4. Establish a preliminary conceptual model for Mindfulness-Based Recovery from Stroke (MBRfS), based on themes that emerged in the process of MBSR curriculum adaptation.
5. Illustrate the conceptual model and its potential contribution via a series of autoethnographic vignettes of the author’s direct experiences.

## 2. Materials and Methods 

### 2.1. Overview of Research Design 

The research design of this study incorporated an iterative and non-linear process of MBSR curriculum content review, reflection utilizing autoethnography, and thematic analysis, all of which results in the proposed curricular adaptations and early development of a mindfulness-based stroke recovery model. More specific steps included: Literature review and synthesis of key elements of the MBSR program, review of the author’s personal documentation of stroke recovery, engaging in autoethnographic reflection and subsequent thematic analysis, identifying novel, critical MBSR adaptations for stroke survivors, drafting the proposed adaptation of the MBSR framework and curriculum, additional thematic analysis to the proposed MBRfS curriculum, and editing the highlighted vignettes from the author’s journals to further illustrate the components of MBRfS. 

### 2.2. Mindfulness-Based Stress Reduction (MBSR) Curriculum Adaptation

The first step in the study’s design involved careful review of the MBSR curriculum to extract key elements by week, attitude, and practice. These elements were synthesized into tabular format and provided the framework upon which all adaptations were proposed. The theoretical foundations of MBSR are a combination of mindfulness training and psychological models of stress perception, with emphasis on the physiological impact of chronic stress related to chronic illnesses. Kabat-Zinn [1] synthesized a model that integrates contemporary theories of stress physiology and stress perception with Eastern wisdom traditions within Buddhism and Yoga, using professional terminology, fitting for a secular, Western context. Mindfulness instruction includes a strong commitment to home practice (approximately 45 min per day) which consists of listening to audio-recorded verbal guidance through a variety of formal mindfulness meditations. Formal mindfulness meditation practices include a body scan meditation, walking meditation, gentle yoga movement, and seated meditation practices. Research demonstrates that the combination of skills offered in MBSR training reduces patient stress and overall levels of distress related to illness and pain [2]. 

While the emphasis of the program curriculum is experiential learning through the direct practice of mindfulness meditation, there is also a strong psychoeducational component. The formal mindfulness meditations practiced are primarily a body scan meditation (lying down), walking and seated mindfulness meditation practices, and gentle yoga sequences. Psychoeducational elements include mind–body models and theories of stress, stress perception and coping, and interpersonal communication skills. 

MBSR training participants are encouraged to establish and clarify their intentions for initiating and continuing the program across the eight weeks, reaffirming a commitment to self-responsibility and their health. Through the ongoing practice and cultivation of mindfulness skills, participants may strengthen the natural capacity for self-awareness and focus attention deliberately, creating a sense of “witnessing” direct experience of their body, physical sensations, thoughts, and emotional states. Furthermore, the curriculum and ongoing practice of mindfulness meditation strengthens the capacity for self-regulation of emotional states, enabling participants to “pause” and choose to respond to a stress trigger rather than react in a patterned, habitual way [2].

Due to the engagement and deepening of mindfulness skills, participants learn experientially that the fight-or-flight response can be diminished, allowing their bodies to return to a state of relative homeostasis more efficiently. This allows for an overall sense of stress management and symptom control arising from stress reactivity in the body. The perception of what participants considered “stressful” often shifts due to the depth of self-awareness combined with participant education of fight-or-flight response and stress perception. Major theories on stress perception and interpersonal communication skills to support growth and increase capacity to integrate mindfulness skills in everyday lives are taught and emphasized. 

For each class, there is an assigned corresponding “home practice” requiring a commitment of 45 min, six days per week. There is also a day-long silent “retreat” that takes place near the end of the program. Home practices consist of listening to guided formal practices introduced across the series of classes and recording events and communications considered “pleasant” or “unpleasant.” Participants keep a log of thoughts, feelings, and physical sensations that reinforce and deepen their capacity for mindful self-awareness. Participants are encouraged to become more aware of affect regulation and the capacity to tolerate events they consider “unpleasant” or painful and to bring attitudes of kindness and compassion toward themselves and others. This is a component adapted from the Buddhist tradition of Metta or “Loving-Kindness” meditation and is introduced later in the program. 

The MBSR curriculum emphasizes the value and inherent dignity of the human experience. The overarching impact of the program is essentially a transformation in perception, refining the attitudinal lens through which we choose to experience our world. It is as much a shift in our way of being as it is a change in what we are doing. The formal practices and unfolding of the curriculum each week are offered with reminders and infused with the attitudes. The “what” of MBSR are the formal practices, and the “how” of MBSR points to the attitudes. 

Using this framework, curricular adaptations specific to stroke recovery were drafted and summarized for each week. Proposed adaptations represent content and themes that emerged through the process of literature review and autoethnographic reflection as the suggested MBRfS model, in addition to the overarching principles and themes that emerged through the iterative process of reflection and modification. This included a detailed review of the author’s reflections during the process of stroke recovery (autoethnographic method described in Section 2.4). 

### 2.3. Autoethnography to Identify Themes and Illustrate the Framework for the Proposed Model

Qualitative research utilizes a variety of data, such as interview, case studies, and personal narratives, to describe and perhaps explain meaning within adverse moments in life [43]. Autoethnography is a practice of inquiry that analyzes the autobiographic narratives of the researcher as the primary data [44]. Common approaches for qualitative analysis of autoethnography are narrative and thematic analysis, along with applied social constructivism. Autoethnography can be viewed as a challenge to traditional academic research, specifically those rooted in positivist and post-positivist paradigms [45,46]. This qualitative approach tends to break the norms of academic discourse, which hide the author by their nature and intent [47]. Conversely, one of the possible merits of autoethnography is in the richness and depth of hearing a direct account of the researcher’s lived experiences.

This preliminary study utilized aspects of the qualitative research method of autoethnography for the purpose of supporting a mindfulness-based support model for stroke survivors. This study emphasized the strengths of qualitative research, consisting of narratives of the author’s stroke recovery experiences, and the specific ways she was able to access and apply the mindfulness skills and attitudes taught in MBSR. 

The narrative vignettes offered are written in the first person, and the author shifted to a first person voice where appropriate when introducing and framing the vignettes, which consist of excerpts from the author’s journals and adaptations of memories stored until it was also physically possible to hold a pen and write in a journal (e.g., photographs and dictation kept on a smart phone). Analysis techniques include a combination of narrative and thematic analysis for the specific purpose of illustrating the possibilities within a mindfulness-based curriculum to support stroke recovery.

The rationale for bringing in autoethnography as the research method to support the introduction of the MBRfS model is that it gave the author, as an MBSR teacher and stroke survivor, the chance to offer lived experience as a focus and impetus [48]. The author’s story, in this case, is the most genuine and relevant place to begin. As Goodall [49] asserted, “If we are willing to study others, we ought to be equally willing to place ourselves, our lives, our families, under the same critical scrutiny” (p. 110). This study incorporated the data analysis elements of an autoethnographic account as a stroke survivor facing the challenges of stroke recovery, including the process navigating the healthcare system and way she utilized her mindfulness meditation skills. The author’s intention in sharing excerpts of her story is not to express a linear or complete factual account of what happened, but rather to communicate the implications for proposing the MBRfS model in hopes it can be used to serve others in the way she ascribed to her experience (thus far) in stroke recovery.

This research study was guided by evaluative questions framed in the first-person for the author to address:

1. What is my lived experience of stroke and stroke recovery (up to this point)?
2. How has my stroke recovery been impacted by my prior MBSR training (first as a participant and later as a teacher)?
3. How did my mindfulness skills influence my navigation of the healthcare system and interactions with healthcare providers?
4. What effects did MBSR training have on my choice to view my stroke and the recovery as a journey and pathway to greater wholeness?
5. How does MBSR continue to contribute to my perceptions of physical health and emotional well-being in stroke recovery?
6. How have I adapted the MBSR model to best serve my own stroke recovery and perhaps also serve other stroke survivors?

### 2.4. Emergent Themes and Principles as a Conceptual Model for Stroke Recovery: MBRfS

After completing the task of clearly identifying the modifications to the MBSR framework and curriculum, thematic analysis and content review were conducted on the modifications (within the context of the larger MBSR framework). This included an inductive approach to theme development and coding [50]. After codes were grouped into larger themes, they were synthesized with the autoethnographical themes to propose a unified model. 

## 3. Results

### 3.1. Mindfulness-Based Recovery from Stroke (MBRfS): The Proposed Conceptual Model

**Figure 1:**
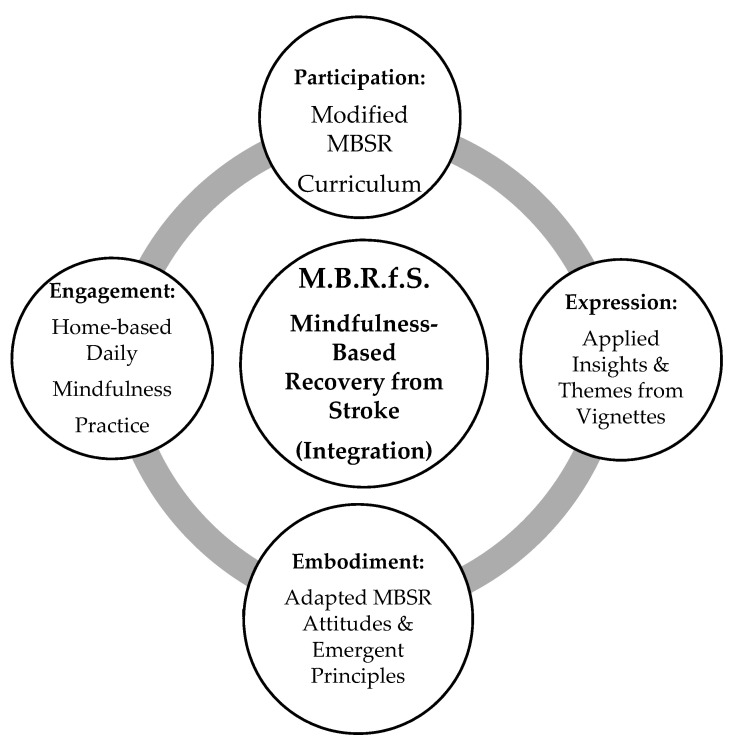
Preliminary conceptual model for Mindfulness-Based Recovery from Stroke (MBRfS).

The introductory MBRfS model offers a starting point for future research studies to refine and develop effective specific mindfulness-based approaches to support those whose lives have been impacted by stroke. The summary figure (Figure 1) depicts the major elements and the iterative, circulatory relationships amongst four dimensions which are integrated as the MBRfS model. 

Each of the four dimensions will be addressed, but they are not intended to be offered in a list of importance, or in linear fashion. Each dimension informs all other dimensions as well as informing the integration of the proposed model. There is an associated quality of action (both of being and doing) for each of the four model dimensions, as follows:

1. Participation: Taking part in the modified eight-week MBSR curriculum.
2. Embodiment: Intention to practice and strengthen the mindfulness-based attitudes and emergent principles (ways of being).
3. Engagement: Commitment to following a home-based daily mindfulness practice during the eight weeks of classes.
4. Expression: Reflect and express personal insights of the stroke recovery journey in the spirit of the author’s autoethnographic explorations and thematic outcomes.

### 3.2. Modifications to MBSR Curriculum (Participation)

Modifications to the Kabat-Zinn traditional framework for MBSR curriculum are presented in the context of the proposed model alongside a synthesis of the original content. To address the scope of the content, the framework was separated into three broad components: Specific weekly curriculum content outlines, traditional attitudes of Mindfulness-Based approaches, and formal mindfulness practices offered in weekly classes and in daily home routines. 

In Table 1, the flow of the traditional MBSR eight-week curriculum is summarized in the first two columns [1]. Specific practices are detailed in association with the eight-week class structure. 

Some of the most foundational modifications to the eight-week class design include:

- Budgeted time for frequent collective and individual rest breaks.
- Shorter versions of the formal mindfulness practices.
- Modifications for all physical movement practices to allow for varying degrees of abilities and energy levels.
- Earlier introduction of modified versions of Mindful Walking as sensory support for walking.
- Offering many versions of shorter practices with easy access for home practice (e.g., MP3, weblinks, CDs).
- Offering summary handouts to support memory for each class and video links to watch or listen to between classes.

### 3.3. Expanded MBSR Attitudes and Overarching Themes of MBRfS (Embodiment)

Table 2 highlights the foundational attitudes of mindfulness: they offer us specific qualities of how to be as we begin a formal relationship in practicing mindfulness. The traditional MBSR attitudes are listed in the first column and the suggested expansion or emphases of each attitude in the context of stroke recovery are offered in the second column. 

Each attitude complements and strengthens every other attitude: it is a highly inter-relational embodiment in the “how” of mindfulness skills. The teacher encourages the embodiment of mindful attitudes by modeling and highlighting examples of the attitudes throughout the curriculum. Sometimes, the teacher will identify the attitudes as they emerge experientially through classroom engagement, and at other times, the teacher will choose to share didactic content. Both instructional approaches are intended to complement and support the other. Related attitudes and principles offered in the proposed model also include:

- Recognition of loss (body feeling, capacity, identity, and self) and the role of grief.
- Non-linear experiences of stroke recovery.
- Attention to change, including progress in strength, flexibility, mobility, and speech.
- Life-long gains in stroke recovery, acceptance that stroke recovery never officially ends.
- Challenges in communication (connected to interpersonal elements of MBSR).

### 3.4. Home-Based Daily Mindfulness Practice (Engagement)

The formal mindfulness practices are summarized in Table 3 and comprise the elements to create a regular home practice routine for class participants. The modifications for each practice are designed to offer the most flexibility and encouragement to stroke survivors, with the ongoing invitation to commit just a few minutes each day to formal mindfulness practice (to minimize frustration or overwhelmingness). As each week of the series of classes meet, the participants are introduced to new variations to choose at home. Each class includes opportunities for participants to share successes and challenges, as well as to ask for support in building a mindfulness meditation home routine.

### 3.5. Autoethnography

*It seems most fitting to first offer reflection on what it is to write this academic paper three years after a major stroke, this act of sharing my thoughts by translating them to paper. I feel a mix of gratitude for the returning abilities and some frustration for the amount of time and combination of strategies the process requires. I take a slow breath and jump into the task, assessing what is needed. Prior to the stroke, I could accurately type almost to the pace of my thoughts, fingers flying across the keyboard in a stream-of-consciousness pace. Now, three years post-stroke, I type using a combination of my non-affected (non-dominant) hand, the pointer finger of my stroke-affected (dominant) hand, and the dictation function, which can listen to my spoken words and transcribe.*

*I find that a dance can occur when I work patiently with whatever shows up at any given moment. Sometimes, my speech is punctuated with stuttering and word searching, which makes dictating more frustrating and less productive. I have also learned to write my thoughts longhand, which sometimes supports better dictation, or I return once again to my slow, modified typing. Sometimes I forget how to spell words, I am not able to envision the words in my mind’s eye, so I also keep my smart phone nearby to dictate the word I want. When I then can see the proper spelling, I recognize it instantly. I gently remind myself that this deficit will continue to improve as I maintain my daily cognitive rehabilitation routine, practicing with a spelling application on my electronic tablet.*

*I return to the concentration of writing, over and over, taking short breaks as needed. I write the first draft with many words spelled phonetically, not worrying beyond that point until I can go back and update for spelling and grammar. And so, my words and intentions gradually come together to be shared with another, whether in an email or a manuscript. A few sentences later, frustrated by the keyboard that used to be so fluid for me, I pick up my cell phone to use the texting and autocorrect that I now find easier. I move along, slowly, deliberately, patiently, as if I have nowhere else to go and I have all the time in the world. The snail has become my symbol and an animal totem to remind me to go slow.*

*I share this as an immediate and simple example of the way I approach life and each cognitive and fine motor task since my experience of a major ischemic stroke in the summer of 2017. At that time, I was a daily runner, avid fan of the Mediterranean diet, and a long-time meditation practitioner and teacher. I did not appear to be at risk for stroke. With low blood pressure and ideal cholesterol levels that caused my doctor to ask with enthusiasm what my diet consisted of, I did not have this particular worry on my radar. And yet, an internal carotid artery on the left side of my face spontaneously occluded and in an instant, my life changed. I became a stroke survivor.*

*The image and teaching metaphor of a tsunami has come to me many times as I attempt to express my personal experience of stroke and these relatively early years of recovery. Before a tsunami hits, there is often a receding of the water along a coastline, a calm before the storm. This is the stage when animals and wise indigenous people can read the signs and head for higher ground. I knew something was not right with me, but I easily dismissed it as jetlag from recent international travel. Next, the tsunami hits, a wall of water slamming with such ferocity to destroy in its path, creating confusion, panic, and devastation. The artery split, and the “wall of water” hit, cutting the blood flow from my brain, depriving my left hemisphere of oxygen, leaving me instantly without the knowledge of how to use my phone to call for help. The tsunami floods and invades the coastline, drowning and breaking apart everything it touches. After the trauma, the water slowly recedes back into the ocean, but the landscape will never be the same. Some parts of the landscape are forever lost, unrecognizable. There is a new vigilance when looking toward the horizon for the possibility of another treacherous wave. Some aspects of the terrain will be repaired, rebuilt, and made anew with time and effort. Unexpected moments of beauty and grace, alongside the sorrows, are the discoveries over time only the strength of the tsunami could reveal: all the hidden gifts under the sand that could not be seen before. This process of repair, discovery, and revealing of hidden treasures never ends.*

*Giant waves, calm seas, the treasures of my mind buried in the sand—this is the way I experience stroke recovery. It is a process that is at times, calm and gradual, other times harsh and unpredictable. It is yet another manifestation of living the “full catastrophe” of life. I am a new and humbled student in this curriculum, vulnerable in the sharing of my lived experience. At the same time, I am deeply aware that I am not alone in this journey, which gives courage to persevere and reach out to share my story with the wish that it be helpful to others.*

*As I reviewed my photos, audio reflection, and journals, I held the questions in mind that I posited in the methods, paying particular attention to the ways in which I deliberately, and sometimes instinctively, relied on my mindfulness skills. This includes what I already knew to be true in regards to the value of MBSR. Simply stated, I have no idea how I could be navigating a lifelong, harrowing journey without a grounding in mindfulness meditation and Mindfulness-Based Stress Reduction (MBSR). I cannot imagine that possibility, and I am grateful that I do not need to. I will share the major themes that spoke to me as I reviewed my recorded experiences, which are reflective of the guiding research questions and informed my proposed adaptations to the MBSR curriculum.*

### 3.6. Autoethnographic Vignettes to Inform the MBRfS Model (Expression)

Four major themes emerged from the autoethnographical study of the author’s journal that directly relate to the proposed model:

1. Acceptance: A readiness and psychological agility to accept the stroke event and the sudden impact of experiencing a stroke and acquired brain injury.
2. Navigating Uncertainties: Expressing and navigating the uncertainties of stroke recovery (both personally and in relationship with healthcare professionals) with mindful awareness and self-responsibility for my recovery outcomes.
3. Somatic Wisdom: Trusting the inherent wisdom of the body and mind to serve metaphorically as stroke recovery “guides” and teachers to accompany stroke survivors on the journey.
4. Meeting Complexities with Compassionate Dignity: The growing capacity over time to integrate (“turn toward”) complex post-stroke emotions, such as fear, grief, vulnerability, and frustration, with greater self-compassion, dignity, and sense of wholeness as a human being.

Each theme is supported by a reflective vignette below. Specific components referenced in the methodological approach included emphasizing the experience of stroke recovery as a lifelong journey, taken on with the spirit of a given mission or odyssey. The acceptance of the journey requires embracing the reality of ongoing doubt, fear, and frustration, while choosing to meet the experience with dignity and courage, which is bolstered through the anchoring to the themes of Acceptance, Navigating Uncertainties, Somatic Wisdom, and Meeting Complexities with Compassionate Dignity. Resiliency in the face of ongoing challenge and adversity is the meta-theme, with its presence woven throughout the narratives.

**Theme** **1.** *Readiness and agility to accept the experience and outcomes*

*Before the stroke in July of 2017, I was participating in a mediation retreat with Jon Kabat-Zinn in Austria. I arrived a few days early and spent each morning hiking the razorback trails of the Alps. I remember how strong my legs were and the way the fresh air hit my face, filled my lungs. The theme of the retreat included a focus on bringing our whole selves, our hearts, and deepest presence into all aspects of life, no matter what. I remember Jon reminding us over and over that the real curriculum is **life** itself, and that we can dedicate our mindfulness practice to meet each new moment of life as if we are greeting the next part of the curriculum. In this way, we learn to fall in love with life itself. I left the retreat feeling renewed and courageous, open-hearted and loving.*

*A week later I experienced the stroke in the wee hours of the night and was unable to alert help. I had a keen awareness, a witnessing of sorts, even in the midst of chaos and confusion. Realization emerged that help may not come in time. I remembered telling myself, “Stay calm. Whatever is happening, it is the curriculum; your heart is here, it is all okay.” I felt the words to be true, even as I witnessed the gradual undoing of my abilities. I was able to remain calm, even though I was unable to move or speak. I was stuck on the floor near my front door, unable to do anything else but remain patient and calm until, at last, help arrived.*

*From the moment I arrived in the emergency room hospital, though I rode waves of fear and confusion, I knew I had experienced a stroke. I was paralyzed with the exception of the left upper quadrant of my body. I could not speak and I was unable to swallow water or food. Unbeknownst to me, the right side of my face was collapsed. In spite of the shock and surrealistic moments, I felt very aware and accepting of the abruptly changed landscape. While the waves of my life crashed up against itself, I was seeking higher ground with a focused resolve, from the very first moment I was able. I have never stopped.*

*I feel certain that my mindfulness practice supported me in staying grounded and present during the stroke event and in the immediacy of the weeks in the hospital. Before I was allowed out of bed, I was visualizing my feet moving and was practicing “mindful walking meditation”. I used my breath to stay calm, and I practiced the body scan mediation to sense into my body whatever levels of proprioception were available to my brain. Life was rehabilitation, and I felt again the sense that life was offering me a novel, challenging, (and unwanted) curriculum. I persisted, sensing a precious window of spontaneous recovery time when my brain could “bounce back”, it was critical for me to take advantage of that time. I pushed, and I learned quickly that my brain also required frequent rest breaks. When I was unable to sleep, I meditated. A new dance between effort and rest was born.*

**Theme** **2.** *Expressing and navigating the uncertainties of stroke recovery*

*I felt so many moments of connection and shared humanity in the weeks of hospitalization, first in critical care, and later, inpatient rehabilitation in a specialized hospital. I recall the nurse from the Emergency Room who came to the Critical Care Unit to visit me. I did not recognize her, but I remembered her voice and started crying when she asked, “Can I please hug you?” She was the first person to care for me after the ambulance delivered me into her steady hands and I remembered her comforting, strong voice.*

*The third day my neurologist visited my room. I remember his soft eyes and kind voice. He sat down, held my hand, and he explained the cause and extent of the stroke. He then paused and said, “This recovery is the hardest thing you will ever do in your life. How you meet it will be the greatest predictor in the outcome. It is largely up to you.” I felt the weight of the words but more the gratitude for his honesty and his kindness. Even now, it brings tears. I knew the lion’s share of responsibility was on me, but I was not alone.*

*There are many other moments I can share of a healthcare professional choosing to be human and express sincere kindness and empathy. My recreational therapist introduced herself and urged me to quickly put on my gym shoes so we could meet our session objectives. She paused to notice me staring down at my shoes, clueless how to tie them. My eyes filled with tears and I felt a childlike sense of vulnerability, of overwhelm. She knelt at my feet and taught me a teaching rhyme to remember how to tie the laces, and I learned how to tie my shoes with one functional hand. She abandoned the treatment goals to be a healing presence instead. My heart swells even now to remember her gentle kindness. Later, when she learned I could play music, we visited a room with a piano: she had already placed a children’s piano book on it for me. If I watched my hand and told my fingers to move, slowly, I was able to play a few lines from Beethoven’s “Ode to Joy”! What a moment that was, to smile and laugh with her at the piano that day!*

*Not all communication was helpful or kind. A physician with a bow tie came to my room and promptly assumed I was abusing my health. He lectured me to stop, “Smoking and drinking,” knowing little about me. Fortunately, I practiced patience (though I lacked the language skills necessary to defend myself at the time had I chosen indignance). However, when the time came for me to be released, I requested to see him again. I offered him some professional feedback, relying on my interpersonal mindfulness skills. I had hopes that he would be willing to truly see and hear me. I intended to speak clearly on behalf of all his patients before he could succumb to the next urge to lecture. It was worth the try.*

*I also was quite feisty when I was told by my caregivers about the inevitable “plateaus” and supposed limits of stroke recovery. I refused to work with those individuals, however well-intentioned they likely were. I dismissed them from my team without hesitation. Looking back, I may not have won a popularity contest at the hospital. Sometimes, we have to be fierce advocates and not “good” patients—it was never meant to be unkind or personal. I knew enough in my own training and professional background about neuroplasticity and the possibilities for the brain to heal and rewire itself over time with proper support. It was essential for me to be surrounded with encouraging and progressive rehabilitation team members. Above all, I knew I was ultimately responsible to “lead” my team and advocate for myself to the greatest extent possible.*

*Above and beyond, I was a keen observer of the level of stress and suffering that existed within the hospital, not only for the patients, but the staff as well. My day nurse and I talked about stress management. I taught her the STOP acronym (Stop, take a Breath, Observe, and Proceed) to remind her to pause and breathe. Often, we did this together. Before she left her shift, she would say goodbye, resting her hand on my forehead for a moment in a comforting way to wish me goodnight.*

*On a broader level, I remember wishing that all the timed trials of task assessments would have included mindful pauses (both for me and the therapist). Seeing the dysfunction of the system and the strain it created on healthcare providers was sobering, but it comingled with the great kindnesses I also received. I wished I could do something to help alleviate the tremendous suffering I witnessed at the hospital, for the patients and their visitors as well as healthcare providers. I understood at a deep level the degree of uncertainties we were **all** living with, day by day, moment to moment. I remain humbled and in awe of the skills and strengths required to cast light on this journey; even in the darkness of doubt, there is healing that comes through accepting the un-knowable.*

**Theme** **3.** *Trusting the inherent wisdom of the body and mind*

*Unexpectedly, my body has taught me more about the depth and brilliance of somatic intelligence since the stroke, more than I even imagined possible. The first moment I awoke to this aspect was a sunrise one morning in the rehabilitation hospital. I had a beautiful view from my room of a cathedral, and the morning sun was reflecting off a tall steeple. My head was turned toward the window, noting the peace and beauty, but I awoke feeling frightened, lonely, and sad. I started to weep. Then, I felt a comforting touch on my non-affected arm; someone was gently rubbing my forearm and patting softly, as if to console. I felt soothed, but I was surprised by the sense that someone was there to comfort me. I looked away from the window and down to my arm. I was stunned to see that my stroke-affected right hand was actually my comforter! I watched my right-hand touch and soothe my left side, as if to say, “All is well. Take heart.” My right side was slowly moving again, though even now, it is still largely numb. But I felt whole in that moment… complete, strong, able, and more than anything else, I felt a trust in the wisdom of my body.*

*Having practiced stroke recovery for three years now, I have come to have a deep trust in something I know I can never fully understand. I rarely feel lonely, even when I am alone. My body knew, likely for many years prior, that one of my internal carotid arteries was growing in a corkscrew pattern and would eventually dissect. The reason I am alive is because my body had a plan well in advance: it had grown a web of collateral arteries, created in advance to take over when the main artery occluded. My body knew what to do to prepare me for the stroke.*

*Each day I learn about the limits, when to challenge and when to rest. I know the energy expended on emotions and how deeply emotions are expressed, resonating throughout the body (with or without the accompanying cognitions). I think less and trust my body more. The home of each visiting breath, my body has become a companion and wise teacher. By collaborating in close partnership with my body, I have become more curious, compassionate, protective of my dignity, and sometimes, even, brave.*

**Theme** **4.** *Capacity to integrate complex emotions with self-compassion and sense of wholeness*

*What my neurologist told me has been true, at least so far: recovery from a stroke has been the hardest experience to endure. I am still in the relatively early years of a stroke recovery journey and I have experienced very pronounced outcomes so far. The marvel to me, most recently, is the non-linear sense of this journey. I am still feeling grief, at times bewilderment, anger, fear, and a host of other challenges. My fluctuating confidence in navigating the shifting emotional landscapes is a fresher aspect of the “curriculum”. Each day poses unique challenges and, when I am well-rested, I can appear to the world as if I never had a stroke (especially to those who did not know me prior). I have learned how to compensate and can often anticipate where I may encounter a difficulty, and I do my best to make back-up plans. For example, If I need to drive somewhere, I gauge my energy to make sure that I have the neurological battery life to find my way back home. If I assess that my brain may not endure, I rely on ride-sharing services or I ask for a ride (or I may choose to stay home). This can be frustrating, but I start with observing my ability to make breakfast: I do it in a sequence each morning, and noticing the relative ease or difficulty (and my emotions rising around the preparation) give me a good sense for how to best approach the day. I am the guardian of my brain’s battery life: I am responsible for advocating and caring for it. Every morning is the first of the day’s lessons in beginners mind and acceptance.*

*To serve as another example, after a work meeting or a visit with a friend, I will take notes as soon as possible—I have learned that I will forget major details… a lunch visit with a friend can feel like a lost dream only a few hours later. I have learned to anticipate and work with these current limitations. I am still challenged by the need to explain to others, to assert myself to ask for what I need, and to experience the emotional vulnerability. I sense the eagerness of friends and family to feel reassured that I am “better”. I want to reassure, and often do, but I also can feel quite invisible and, ironically, more isolated as a result. I remind myself that, again, this all points to a deeper learning: it is the unfolding curriculum. Even more than practicing patience, I become more adept at forbearance and finding compassion for all of us. I am not the only one impacted by the stroke. I am profoundly aware that we are doing the best we can with what we currently know, day by day, moment by moment.*

*As I was trained to do though meditation, I pay careful attention to my thoughts. For the first few months of recovery, I noted how very quiet my mind was due to the brain injury. Ironically, it was also peaceful and helpful to have less intellectual chatter. As my brain heals and forms new connections, I am experiencing more cognitions and complex emotions. It is as if I am a new person experiencing a very different life. Even now, I feel I fall short to explain the challenge of psychological navigation in this aspect of stroke recovery. Regardless, the journey continues.*

*I return to the tsunami metaphor. The identity of a stroke survivor is swept away and we are forever changed. One of the most important aspects of my prior training in mindfulness is the awareness that I am not alone in my experience. “I” am still “here”, even as I may seek to grieve and restore aspects of my inner landscape, I also find hidden treasures in the sand, aspects of myself that are new delights. I can hold much more complex and even paradoxical emotions without feeling the need to perceive or resolve imaginary dissonance amongst the kaleidoscope of emotions.*

*I inwardly step back and marvel at the mystery and beauty of the whole thing, this big beautiful “catastrophe of living”. If I can fall in love with life, then surely, I have fallen in love with stroke recovery as part of it. It is a lifelong love affair that demands nothing less than everything. In return, it offers me the world and each moment in it. I am often reminded of a teaching poem in the MBSR tradition:*

*Love after Love by Derek Walcott*

 

*The time will come*

*when, with elation*

*you will greet yourself arriving*

*at your own door, in your own mirror*

*and each will smile at the other’s welcome,*

 

*and say, sit here. Eat.*

*You will love again the stranger who was yourself.*

*Give wine. Give bread. Give back your heart*

*to itself, to the stranger who has loved you*

 

*all your life, whom you ignored*

*for another, who knows you by heart.*

*Take down the love letters from the bookshelf,*

 

*the photographs, the desperate notes,*

*peel your own image from the mirror.*

*Sit. Feast on your life.*

## 4. Discussion

### 4.1. Offering a Proposed Integrated Mindfulness-Based Model to Support Recovery from Stroke

This study utilized an iterative and non-linear process of MBSR content review, consideration of the unique challenges for stroke survivors, reflection through autoethnography, and thematic analysis, all of which results in the early stages of a proposed mindfulness-based stroke recovery model. The hope and intention of this endeavor is, at this stage, hypothetical and aspirational. The potential value of this MBRfS model is intersectional. Minimally, the curriculum may help stroke survivors lower their stress levels and be better able to engage in successful recovery behaviors, and this alone could be a valuable contribution. The impact in reducing or buffering depression is another promising possibility. Mindfulness skills may also raise concentration levels and support restorative, brain-healing rest in recovery from stroke. Further still, the deeper levels of mindfulness-based learning may reveal some deeper insights for healing. Exploring the depth and breadth of the journey of stroke recovery with others and walking the same path can reveal deeper insights. For some participants, MBRfS could offer a source for personal awakening and new discoveries, and over time, perhaps a deeper acceptance of the “full catastrophe” of stroke recovery. The journey never ends. Stroke is both the teacher and the curriculum, and all participants (including the facilitator) serve as both teacher and student, to and with each other on the path toward recovery. The growth and learning can become a blueprint for how we choose to live our lives as whole human beings. 

### 4.2. Curriculum Integrity of MBSR as a Modified Curricular Intervention for Recovery from Stroke

One of the challenges and commitments to the MBSR curriculum is maintaining the essential core aspects of the training while allowing the space to accommodate the collective personality and group dynamics created within each unique constellation of individual participants. In order to maintain quality and assurance for the public to maintain and increase faith in mindfulness-based training, the field must commit to upholding the established protocol in both practice and research. Leaders in the field have suggested four essential ingredients for all mindfulness-based programs: (1) to be theoretical, and in practice, informed by established contemplative traditions as well as healing sciences, (2) to develop a new relational experience with present moment awareness, (3) to provide supportive development of self-regulation skills, and (4) to engage participants in intensive mindfulness meditation training. Remaining elements are open to thoughtful and deliberate adaptation with the intention of a better “fit” across specific populations and contexts, such as varying the program length, structure, and delivery [54]. 

The developmental challenge to curricular fidelity, as MBRfS and other mindfulness-based interventions may increase in popularity, is to continue to rely on the foundational framework as a “map”, while also responding spontaneously to whatever shows up in the classroom. This requires not only tremendous teaching skills, but also deep teacher engagement that demonstrates the embodiment of mindfulness practice [55]. Integrating clearer assessments of teacher skills and developmental growth, such as the Mindfulness-Based Teaching Assessment Crieteria (MBI: TAC), would also be an important component to consider [56,57].

### 4.3. Themes and Challenges Specific to the MBRfS Program

Accessibility and appropriate adaptations are the broadest challenges in offering the MBRfS curriculum. There are immense variations in the deficits for stroke survivors. Fatigue, emotional lability, speech, and expressive disorders are all very challenging to accommodate in a group format. The possibilities of individual programs instead of, or in addition to, group participation need to be considered, as well at the value of inpatient and outpatient programs. A remote learning option (either offered individually or for groups) could outlast the COVID-19 utility and be another way to increase accessibility. Clear participating criteria should be developed, and it may be recommended for individuals to be assessed by a clinical neuropsychologist, both in support of patient safety and to serve ongoing development of participating criteria. An orientation session that would include assessment and an individual interview is advised to guide potential participants very closely, and with an expressed care for their well-being. Ideally, a developmental continuum of this model will evolve so that no one expressing a wish to learn mindfulness skills would be turned away—participation ought not be binary. There are many challenges to explore which will require creative and well-informed solutions, with an interdisciplinary approach. 

### 4.4. Future Directions

There are numerous dimensions that may be helpful avenues for further development and refinement of the MBRfS model. Healthcare providers who serve stroke patients, rehabilitation specialists, caretakers, and others in support service roles may benefit, not only for the stress reducing and resiliency promotion of practicing mindfulness meditation, but also to increase empathy and connection when working in partnership with stroke survivors. In addition to those in stroke recovery, patients who are assessed as “at-risk” for experiencing a stroke (e.g., transient ischemic attack, hypertension) may benefit, both as a preventative step in reducing stress, and by participating in a supportive preparation, if a stroke event should occur. 

### 4.5. Limitations 

The fundamental limitation of this study is its reliability: we cannot know whether the analysis and conclusions could be transferable to others in this or a similar situation. The possibility of author bias is another limitation, especially given the few studies in this area for comparison or theoretical support. Given that every stroke and its subsequent effects are unique, great care must be taken in generalizations or replication of stated outcomes. It is worth noting that the author received her mindfulness training and had a longstanding mediation practice before experiencing a stroke. In contrast, this study proposes that the MBRfS model could be similarly helpful for someone to learn after surviving a stroke. 

### 4.6. Implications for Future Studies

This study is a small but potentially significant first step. Research that seeks to refine empirically valid interventions, teacher training guidelines, and effective curriculum adaptations will support success in offering mindfulness-based interventions for stroke survivors and their caregivers. Future studies will potentially guide educational models and resources for healthcare providers practicing in these settings. As with all intervention research, care must be taken to uphold the integrity of the intervention to ensure that reasonable results can be replicated, while incorporating the competencies of the teacher, along with careful monitoring of intervention delivery [58].

## 5. Conclusions

The story of Zorba the Greek reminds us to embrace and love the “full catastrophe” of being alive and human, no matter our current circumstances. According to Dr. Jon Kabat-Zinn, founder of MBSR, “As long as you are breathing, there is more right with you than wrong with you, no matter what is wrong” [1] (p. 26). This quote is continuously shared with MBSR participants at the first meeting and throughout the training period to remind them that they are always “more” than their symptoms and suffering. From this viewpoint, participants learn to experience their bodies and minds as already whole, where the capacity and container for living their lives deepens and broadens over time and with the deepening of their mindfulness meditation commitment. Their experience becomes their most powerful teacher, their practice their greatest ally in the daily challenges of living. Above all, they learn from each other that they are never alone, no matter their experience. A strong sense of interconnectedness and humanity often arises during the workshops as a result of this collective, experiential education. 

*May all beings be healthy and well.*

## Figures and Tables

**Table 1:** Comparing traditional elements of the MBSR program with the proposed Mindfulness-Based Recovery from Stroke (MBRfS) program (class structure and outline).

MBSR Classes ^1^	Themes and Formal Mindfulness Practices	Home Practice Invitations	Suggested Modifications for MBRfS Classes
Week One: Introductions and Establishing Group	The Seven “Attitudes of Practice”. Introduction to definition of mindfulness. Mindful Eating Experience. Body Scan Practice.	Body Scan Meditation. Reminders of Attitudes. Eat a mindful meal. Noticing informal mindfulness.	Introductions and establishing the group. Recognizing that we are all on the same journey, but no two journeys are alike. Introducing the seven attitudes through the lens of stroke recovery Introduction to the body scan practice, with participant encouragement to “sense into” the stroke-affected side. Introduce a seated version of Mindful Walking practice, encouraging sensing into feet and the act of placing footsteps. Movement can be imagined if not possible on affected side. Honoring the need for rest. Sensing when brain is approaching neurofatigue and knowing when to slow down and take breaks.
Week Two: Perception and Stressors	Body Scan Meditation. Standing Yoga/Mindful Movement. Reflections of perception and stress. Introduction to seated Awareness of Breath (AOB) practice.	Body Scan Meditation. Awareness of Breathing Meditation. Fill out “Pleasant Events” calendar. Informal mindfulness.	Balancing compensation techniques with the importance to challenge current deficits in stroke recovery. Invitation to explore the unique stressors for stroke survivors. Very brief introduction to AOB practice, incorporated into body scan and offered as a very abbreviated “brain break” in working with neurofatigue. Mindful Walking (or seated walking) and modified Chair Yoga for standing postures. (imagining the movement if unable on affected side).
Week Three: Pleasant and Unpleasant experiences	AOB practice. Lying down Yoga/Mindful Movement Practice. Discussion of “Pleasant Events” homework.	Body Scan Meditation. Mindful Movement. Fill out “Unpleasant Event” Calendar. Awareness of Breathing Meditation.	Short practice sessions, including very short (3 min) breathe awareness practices as “brain breaks”. Teaching acronym: STOP (Stop, take a breath, Observe, and Proceed). ^2^ Mindful Walking (Standing or sitting options/modifications). Combine body scan with short sequence of lying down movements (imagining the movement if unable on affected side). Focused expression (invitation) on the experience of a pleasant events in the midst of stroke recovery. (“As long as you’re breathing, there’s more right than wrong happening with you, no matter what’s wrong.” ^1^) Hold this in your mind as you are able, expressing it either verbally or non-verbally.
Week Four: The Stress Cycle and responding to stressful events	Seated Mindfulness Meditation. Exploring how thoughts can contribute to stress. Responses to “Pleasant” and “Unpleasant” Events. Standing and lying down Yoga practice	Body Scan Meditation Yoga/Mindful Movement. Seated Mindfulness Meditation. Noting stress reactions and behaviors.	Short practice sessions, including very short (3 min) breath awareness practices as “brain breaks”. Mindful Walking and stretches (Standing or sitting options/modifications), Short practice sessions, including very short (3 min) breath awareness practices as “brain breaks”. Mindful Walking (Standing or sitting options/modifications). Combine Body Scan with short sequence of lying down movements, imagining the movement if unable on affected side). Combine Body Scan with seated meditation guidance. Invitation to express the experience of a pleasant and the additional element of unpleasant event in the midst of stroke recovery (e.g., allowing others to help, experiencing vulnerability as a stroke survivor). Hold this in your mind as you are able, expressing it either verbally or non-verbally.
Week Five: Moving from stress reactivity to responsivity.	Seated Mindfulness Meditation. Stress Reactivity vs. Stress Responsivity: What’s the difference? Introduction to Mindful Walking.	Alternate daily between Body Scan, Seated Meditation, and Yoga/Mindful Movement. Complete the “Difficult Communications” Calendar. Find informal moments to respond with responsive awareness.	Invite seated mindfulness practice lying down, standing, or sitting, encouraging change of posture as needed by sensing what the body needs and adjusting. Challenge, but not overwhelm. Mindful Walking (Standing or sitting options/modifications). Continue discussion of stressors related to recovery and possibilities for responding to stress rather than reacting. Positive neuroplasticity and the negativity bias. Challenging stressful thoughts and behaviors surrounding stroke recovery. Naming the common pitfalls of self-defeating beliefs about stroke recovery. Invitation to explore the challenge of navigating new and complex emotions, fear and developing courage. Teaching acronyms: RAIN (Recognize, Accept, Investigate, Nurture).^3^
Week Six: Interpersonal mindfulness.	Seated Mindfulness Meditation. Mindful Walking Practice. Discussion of Difficult Communication Calendar and interpersonal stress.	Alternate daily between Body Scan, Seated Meditation, and Yoga/Mindful Movement. Mindful walking once this week. Awareness of interpersonal mindfulness and responding in communications with awareness. Informally pause and notice your breath throughout the day.	Invite seated mindfulness practice lying down, standing, or sitting, encouraging change of posture as needed. Challenge, but not overwhelm. Mindful Walking. (Standing or sitting options/modifications.) Invitation to express the experience of difficult communication. (Challenge in communication with caregivers, family, friends, and healthcare providers about your experience and needs in stroke recovery.)
Day of Mindfulness.	A Day of Silence during the weekend between class six and class seven to practice the formal mindfulness meditations together, including a mindful meal. Loving Kindness Meditation. Lake or Mountain guided imagery meditations.	Continue homework from Week Six.	Include rest breaks and a dedicated area for participants to rest/sleep as needed through the day. Loving Kindness meditation. Lake or Mountain guided imagery meditations. Provide simple and easy to eat, nutritious snacks for breaks (e.g., protein drinks, cut up fruit, nuts). Invitation to reflect on the role and relationship to rest as restoration and as an important companion for recovery.
Week Seven: Developing a home practice and continuing interpersonal mindfulness	Seated Mindfulness Meditation. Reflection on the Day of Mindfulness. Mindful Communication. Loving Kindness meditation. Lake or Mountain guided imagery meditations.	Practice with no audio guidance this week. Alternate practices daily (30–45 min) choosing a combination of Body Scan, Walking, Yoga, and Seated Mindful Meditation. Use the audio guidance if this feels frustrating to go without.	Invite Seated Mindfulness practice lying down, standing, or sitting, encouraging change of posture as needed. Challenge, but not overwhelm. Loving Kindness meditation. Lake or Mountain guided imagery meditations. Invitation to express, reflect, and acknowledge: Who am I now? How have my relationships been impacted? What do I need to care for my emotional health?
Week Eight: Coming Full Circle	Body Scan Meditation. Review and taking stock. Reflections and discussion. Closing the circle and saying goodbye.	Return to home practices in any combination, with and without guided audio recordings.	Combine Body Scan with guided Loving-Kindness practice, emphasizing Loving-Kindness for our bodies. Allow space for expression and review. Intention setting: The importance of the fundamental mindfulness attitudes and self-compassion to help us stay on the path. Practice Loving-Kindness. The end of this week creates a space filled with potential emotion, including fear. The eight weeks created a loving, compassionate, and thoughtful container that patients are now leaving. What elements of this transition might need to be considered? Allow space for expression of emotion and grief that may emerge with this ending. Offer a guided meditation that draws attention to the space of transition. Ongoing needed resources, social support, maintaining motivation. Continuing to make life-long gains in stroke recovery, accepting that recovery never ends. Life and rehab/recovery are our teachers and the “curriculum”.
Overarching Practices Across All Weeks (Emphasis on process)	Embodiment of practices and attitudes. Social Support. Commitment.		Classes weekly; Expand availability to include two identical sessions each week. This allows opportunity for missed days or the benefits of repetition and social contact. Create a resting space where participants can easily lie down or take additional breaks as needed.

^1^ MBSR Curriculum adaptations developed using the 2017 MBSR Authorized Curriculum Guide [51], ^2^ STOP technique developed by Stahl and Goldstein [52], ^3^ RAIN technique adapted by Brach [53].

**Table 2:** Introduction and expansion with reframing the attitudes of mindfulness in the context of stroke recovery.

Attitude	Definition of Attitude in Traditional MBSR Context	Suggested Expansion and/or Adaptation of Attitude in MBRfS Context
1. Non-judgment	Practice of not being caught up in our ongoing assessments (both internal and external). Noticing ongoing automatic thoughts of judgments and self-criticism.	Noting self-criticism, judgments, or imposing stigma about having a stroke and efforts toward recovery.
2. Patience	Recognizing that everything unfolds in its own time, some processes cannot be expedited. Patience contains its own kind of wisdom.	Patience in the stroke recovery journey and the length of the time it takes to heal the brain and regain lost abilities, also patience with setbacks during the recovery process.
3. Beginner’s Mind	A willingness to see our experience with fresh eyes, a sense of newness rather than the patterns of our opinions, feelings and cognitions. Staying curious.	Using curiosity in assessing gains and not concluding that a recovery plateau has occurred. Staying open minded to see recovery with new eyes every day.
4. Trust	Honoring and staying in tune with our instincts and our own inner compass for guidance. Relying on our basic goodness and wholeness.	Learning to trust the body and especial the brain to heal and guide us, seeing we are “whole” as stroke survivors.
5. Non-Striving	To back off on the intensity to achieve or excessively pushing goal behaviors in hopes of getting desired results.	In stroke recovery, we must learn when and how far to challenge, but not push into overwhelm or regression.
6. Acceptance	A willingness to see things as there presently are, not as we might wish them to be. Coming to terms with a situation rather than forcing or denying. Not to be confused with resignation or giving up.	Acceptance of experiencing stroke is critical to engaging in recovery. We do grieve, but accept what has happened. Acceptance means we engage more fully in recovery efforts sooner, which maximizes recovery potential.
7. Letting Go	Practice of not grasping on to the things we want, while rejecting that which we do not want. Sometimes saying we can “leave it be” is a first step toward letting go.	For stroke survivors, we are challenged over and over, to let go of what our bodies and identities were before the stroke, while still engaging in recovery.

MBSR Curriculum adaptations developed using the 2017 MBSR Authorized Curriculum Guide [51].

**Table 3:** Formal mindfulness practices for home practice routine and recommended MBRfS adaptations.

Formal Mindfulness Practice	Description in Traditional MBSR Context	Suggested Expansion and/or Adaptation in MBRfS Context
1. Body Scan Meditation	Practiced while lying on back and sensing into the body from toes up through the top of the head, including invitations to breathe into each region of the body. Length of practice is approximately 45 min.	With all practices, offer a shorter version as an option to accommodate fatigue and concentration difficulties. Incorporate simple “sensory support” mechanisms on a person-by-person basis to stimulate sensory awareness and integration between the stroke-affected and non-affected sides, such as holding or squeezing small objects (e.g., a ball or stuffed animal). This may also include laying weighted objects gently across sections of the body, such as a weighted blanket. Guiding the Body Scan, similarly, adapting language to incorporate awareness of the stroke affected side of the body. More time encouraging somatic awareness, especially if there is numbness (very common). Still an emphasis on breathing into the whole body and imagining the body in its totality, with participant encouragement to “sense into” the stroke-affected side with whole body breath and with kindness, recognizing that participant may not have feeling in many places across the body.
2. Mindful Walking Meditation	Practicing a deliberate walking pace that is slow and generally moves in a circle (if practicing in a group) or walking slowly and pivoting back and forth in a short row, focusing on the sensations in the feet and the movement of walking. Length of practice varies.	Offer a sitting version that allows just the practice of lifting and placing feet in the ground, or standing up in one place, placing each foot up and down. Or, placing the non-affected side but imagining the stroke affected foot moving (if there is paralysis on stroke affected side). With all practices, offer a shorter version as an option to accommodate fatigue and concentration difficulties.
3. Yoga/Mindful Movement Sequences	Gentle stretching sequences of either standing or lying down Yoga postures. The emphasis is to practice very slowly as an exploration of breath and inviting movement into the body. Length of practice is approximately 30–45 min (two sequences offered, either standing or lying down postures).	Offer Chair Yoga versions of the Yoga postures. Invite visualization of all movements, with or without the ability to also move physically. Encourage “beginner’s mind” to challenge but not overwhelm. With all practices, offer a shorter version as an option to accommodate fatigue and concentration difficulties. Include invitations for the greatest possible movement in the present moment even if that is simply the extension of a finger or a foot placement (e.g., Finger Yoga). Visualization of exercise can offer benefits even if the individual is incapable of conventional kinds of physical exercise.
4. Seated Mindfulness Meditations	Considered the central formal practice, Seated Mindfulness Meditation is practiced usually on a chair, cushion, or bench, with an upright posture with primary attention on the breath. When the mind is distracted, notice, and return attention gently again to the breath. Formal practice is variable, but the intention is to practice daily for around 45 min (may be combined with other formal practices listed above).	Invite any posture that feels right, and the permission to shift postures as needed. With all practices, offer a shorter version as an option to accommodate fatigue and concentration difficulties.
5. Additional mindfulness meditation practices: Loving Kindness, imagery-based guided meditations (e.g., Lake and Mountain)	Loving-Kindness is a meditative practice from Buddhist traditions which encourages sending good wishes for health, happiness, and well-being, first to oneself and then to others. The Lake and Mountain Meditations are examples of guided imagery which suggest the embodiment of the symbolic qualities that different scenes of nature can teach us.	Invite any posture that feels right, and the permission to shift postures as needed. With all practices, offer a shorter version as an option to accommodate fatigue and concentration difficulties. Adapt Loving-Kindness to include compassion for the body and emphasize aspects of courage and strength with the nature-based guided imagery.

MBSR Curriculum adaptations developed using the 2017 MBSR Authorized Curriculum Guide.
